# An Overview of Depression Pathogenesis: Prevailing Hypotheses and Associated Neurobiological Underpinnings Primarily Evidenced by Rodent Studies

**DOI:** 10.1155/da/1891046

**Published:** 2026-09-22

**Authors:** Bo Zhang, Chuanyu Li

**Affiliations:** ^1^ Guangxi Key Laboratory of Brain and Cognitive Neuroscience, Faculty of Basic Medical Sciences, Guilin Medical University, Guilin Guangxi 541199, China, glmc.edu.cn

**Keywords:** BDNF/TrkB dysregulation, depression, glutamate excitotoxicity, HPA axis dysregulation, MGB axis dysfunction, monoamine deficiency, neuroinflammation, oxidative stress

## Abstract

Depression has emerged as a globally prevalent mental disorder that severely compromises both physiological and psychological well‐being. Its widespread prevalence constitutes a pressing public health concern that necessitates intensified attention and targeted therapeutic interventions. Clinical studies primarily delineate core symptomatology of depression and validate the therapeutic efficacy of candidate antidepressants in clinical cohorts. Complementarily, preclinical animal studies, predominantly utilizing chronic stress‐induced rodent models, decipher the sophisticated molecular underpinnings of depression, and enable primary screening for high‐potency antidepressants and innovative therapeutic regimens. Decades of clinical and preclinical research have substantially facilitated the establishment of diverse pathogenic hypotheses and development of first‐line antidepressant therapies. To date, prevailing mechanistic hypotheses include classic theories regarding hypothalamic–pituitary–adrenal (HPA) axis dysregulation and monoamine deficiency, intermediate mechanisms centered on glutamate excitotoxicity and brain‐derived neurotrophic factor dysregulation (BDNF), and emerging theories of neuroinflammation, oxidative stress, and microbiota–gut–brain (MGB) axis dysfunction. Currently available antidepressant regimens primarily comprise conventional monoamine‐targeted agents and novel glutamate‐modulating drugs (e.g., tricyclic antidepressants [TCAs], monoamine oxidase inhibitors [MAOIs], selective serotonin reuptake inhibitors [SSRIs], and esketamine), which, however, are plagued by inherent limitations and undesirable adverse reactions. Despite substantial advances achieved to date, further in‐depth mechanistic investigations are still warranted to develop novel, safe, and efficacious antidepressants and optimize current therapeutic regimens. This review systematically recapitulates the prevailing pathogenic hypotheses and dissects associated multifaceted neurobiological underpinnings, with key evidence predominantly obtained from preclinical chronic stress rodent models. We outline the defining features of each mainstream hypothesis, and the intricate crosstalk and bidirectional regulatory networks among distinct mechanistic pathways, with a particular focus on underlying molecular signaling cascades, thereby constructing a holistic landscape of depression pathogenesis.

## 1. Background

Depression is a common mental disorder, clinically characterized by core symptoms of depressed mood and anhedonia lasting for no less than 2 weeks, along with the most common accompanying symptoms such as sleep disturbances, fatigue, significant changes in appetite or body weight, impaired concentration, excessive guilt, and suicidal ideation [1–3]. Epidemiological data indicate that depression influences ~6% of the world population, with lifetime risk around 18% and global disability‐adjusted life‐years about 16.4% [4–6]. Consistent with global epidemiological trends, depression prevalence among Chinese adults is ~6.9%, while the disease influence on children, adolescents, and graduate and PhD students has been gradually recognized [7–10]. These epidemiological data demonstrate the high prevalence and profound social impact of depression globally and domestically, rendering it an escalating public health issue that demands targeted prevention and timely intervention. Furthermore, the grim epidemiological scenario underscores an urgent need to further elucidate the intricate pathogenesis of depression in order to develop innovative therapeutics and optimizing existing clinical regimens.

Clinical studies first delineate the symptomatic profiles of depression and ultimately verify the antidepressant efficacy of novel compounds in clinical cohorts. Translational medicine serves as a crucial bridge connecting clinical practice and preclinical research, thereby profoundly deepening our mechanistic understanding of depression, promoting the establishment and optimization of pathogenic theories, and accelerating the development of novel antidepressant therapeutics. Preclinical animal studies, especially those employing rodent models, enable in‐depth elucidation of sophisticated molecular mechanisms and preliminary screening of candidate drugs and emerging therapeutic strategies. Rodent experiments are generally implemented following stringent ethical guidelines, which prioritize animal welfare through rigorous strategies, such as animal reduction and pain alleviation. Multiple rodent depression models have been established via diverse approaches, such as pharmacological interventions (e.g., lipopolysaccharide [LPS] and corticosterone), chronic stress exposure, and olfactory bulbectomy [11–13]. As a predominant clinical risk factor for depression, chronic stress recapitulates key depressive symptomatology in rodents. Chronic stress paradigms satisfy three canonical validity benchmarks for preclinical models, including face, construct, and predictive validity, whose pathological signatures recapitulate clinical depression and respond to first‐line antidepressant treatments [12]. Validated by rigorous preclinical evaluation standards, chronic stress paradigms have been widely adopted in frontline depression research. Various stress protocols spanning pre‐weaning and post‐weaning periods can reliably elicit robust depressive‐like behavior, typically manifested as anhedonia and behavioral despair. Maternal separation (MS) represents the most commonly used early‐life stress paradigm, which interferes with dam‐pup attachment throughout the pre‐weaning developmental stage [12]. Multiple validated post‐weaning stress paradigms, encompassing chronic unpredictable mild stress (CUMS), chronic social defeat stress (CSDS), and chronic restraint stress (CRS), elicit stress responses spanning a full spectrum of mild‐to‐severe intensities [12]. Furthermore, pre‐weaning and post‐weaning protocols can be implemented in tandem, as exemplified by MS combined with the social isolation (MSSI) paradigm, to assess the long‐term impacts of early‐life stress on later‐life stress reactivity [14, 15].

Decades of accumulating clinical and preclinical investigations have substantially deepened our mechanistic understanding of depression and yielded multiple well‐recognized pathogenic hypotheses. Collectively, these etiological frameworks cover the classic theories centered on hypothalamic–pituitary–adrenal (HPA) axis dysregulation [16] and monoamine deficiency [17] and later‐established mechanisms represented by glutamate excitotoxicity [18] and dysregulated brain‐derived neurotrophic factor (BDNF)/tyrosine kinase B (TrkB) signaling [19]. Additionally, emerging theories prioritize neuroinflammation [20], oxidative stress [21], and microbiota–gut–brain (MGB) axis dysfunction [22]. To delineate a holistic landscape of depression pathogenesis, this review aims to systematically recapitulate those prevailing hypotheses and dissect associated multifaceted neurobiological underpinnings, predominantly uncovered by preclinical chronic stress‐based rodent studies—a dimension that remains underintegrated in prior reviews. We outline the defining features of each mainstream hypothesis and the intricate crosstalk and bidirectional regulatory networks among distinct mechanistic pathways, with special attention to their underlying molecular signaling cascades.

## 2. Pathogenic Hypotheses and Underlying Mechanisms of Depression

### 2.1. HPA Axis Dysregulation

#### 2.1.1. HPA Axis Dysregulation in Depression

**Figure 1:**
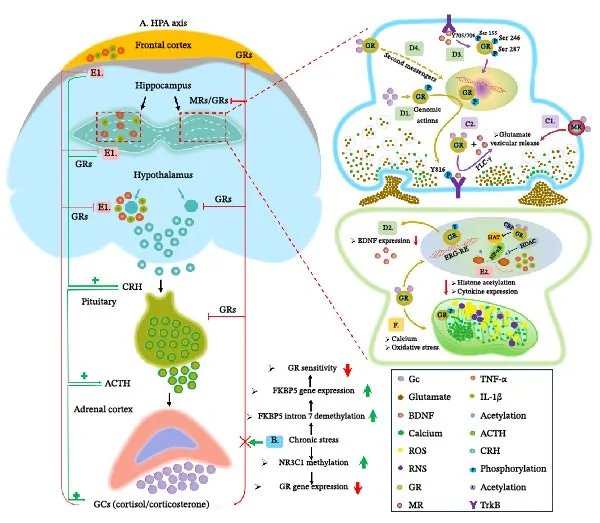
Involvement of HPA axis dysregulation in the pathogenesis of depression. (A) The HPA axis sequentially secretes CRH, ACTH, and GCs. GCs exert negative feedback suppression of upstream HPA activity predominantly via GRs, which are abundantly expressed across the pituitary, hypothalamus, hippocampus, and PFC. (B) Chronic stress disrupts this homeostatic feedback loop: it downregulates GR expression by increasing *NR3C1* methylation and reduces GR sensitivity via promoting demethylation of *FKBP5* intron 7. (C) GC‐activated GRs facilitate glutamate release via two distinct mechanisms: binding to presynaptic MRs (C1) or interacting with TrkB to trigger downstream PLC‐γ signaling (C2). (D) GC‐activated GRs exert reciprocal crosstalk with BDNF signaling: GRs enhances TrkB phosphorylation at the Y816 site via genomic action (D1) or downregulates *BDNF* expression by directly binding to the EGR‐RE located in BDNF exon IV promoter region (D2); BDNF induces GR phosphorylation at serine residues 155/246/287 (S155/246/287) via the intracellular Y705/706 residues of TrkB, thereby remodeling GC‐dependent transcriptomic landscapes (D3). Furthermore, GCs modulate gene transcription through two distinct pathways: GCs directly bind to cytoplasmic GRs and facilitate GR nuclear translocation (D1/2), or indirectly interact with membrane GRs to activate downstream second messenger cascades (D4). (E) The HPA axis exhibits reciprocal crosstalk with the inflammatory system: pro‐inflammatory cytokines potentiate HPA axis activity and GC secretion (E1); GC‐activated GRs translocate into the nucleus and suppress the transcription of pro‐inflammatory cytokine genes, via directly binding to coactivators (e.g., CBP) to inhibit HAT activity, or indirectly recruiting HDAC that reverses histone acetylation and thereby inactivates the NF‐κB complex (E2). (F) Activated GRs modulate oxidative stress via mitochondrial Ca^2+^ signaling. BDNF, brain‐derived neurotrophic factor; CBP, CREB binding protein; EGR‐RE, early growth response protein 1 response element; *FKBP5*, FK506 binding protein 51 gene; GCs, glucocorticoids; GR, glucocorticoid receptor; GRs, glucocorticoid receptors; HAT, histone acetyltransferase; HDAC, histone deacetylase; HPA, hypothalamic–pituitary‐adrenal; MR, mineralocorticoid receptor; NF‐κB, nuclear factor kappa‐B; NR3C1, nuclear receptor subfamily 3 group C member 1; PLC‐γ, phospholipase C‐γ; TrkB, tyrosine kinase B.

To briefly describe physiological HPA axis activity, the hypothalamus secretes corticotropin‐releasing hormone/factor (CRH/CRF), which acts on the pituitary gland to trigger the release of adrenocorticotropic hormone (ACTH) that stimulates the adrenal cortex to synthesize and secrete glucocorticoids (GCs) (Figure 1). Circulating GCs exhibit robust intrinsic circadian oscillations with morning peaks and midnight troughs. Depressive states disrupt this homeostatic rhythm, characterized by a flattened circadian profile, exacerbated diurnal decline, and enhanced nocturnal GC secretion [23, 24]. GCs maintain HPA axis homeostasis via negative feedback suppression on CRH and ACTH secretion, predominantly by binding to glucocorticoid receptors (GRs). GRs are ubiquitously expressed across key brain regions, including the pituitary, hypothalamus, hippocampus, and prefrontal cortex (PFC) (Figure 1). GCs affect gene transcription through directly activating cytoplasmic GRs, which translocate into the nucleus and bind to DNA, or indirectly via activating membrane GRs and downstream second messengers [1, 25] (Figure 1D1/4).

The pivotal role of HPA axis dysregulation in depression has been well documented for decades [2, 16]. Deficient feedback suppression results in HPA axis hyperactivity and sustained elevations of circulating CRH, ACTH, and GCs, a state defined as hypercortisolism that is tightly linked to typical depression [16, 26]. Chronic stress compromises the feedback suppression via epigenetic remodeling. For instance, childhood adversity epigenetically downregulates GR expression and reduces GR sensitivity through enhancing *NR3C1* methylation and *FKBP5* intron 7 demethylation, respectively, thereby perturbing homeostatic feedback and leading to hypercortisolism [27, 28] (Figure 1B1). In contrast, hypocortisolism is characterized by HPA axis hypoactivity and diminished circulating hormone levels, a phenotype strongly correlated with atypical depression [16, 29]. These divergent HPA axis alterations stem from multiple influencing variables, including clinical depression subtypes (e.g., close association between melancholia and hypercortisolism and between atypical depression and hypocortisolism) and methodological discrepancies across preclinical studies (e.g., varied sampling time points and biochemical detection protocols).

#### 2.1.2. Reciprocal Crosstalk Between HPA Axis Dysregulation and Other Pathologic Mechanisms

Interactions between HPA axis dysregulation and other pathogenic mechanisms are predominantly mediated by GRs, whereas mineralocorticoid receptors (MRs) exert partial modulatory effects, as illustrated in Figure 1. GRs and MRs operate either independently or synergistically in a cell‐type‐specific and finely balanced manner [30]. Notably, a previous study demonstrated that astrocytic GRs were more susceptible to stress than neuronal counterparts, broadening our insight beyond the canonical neuronal framework [31]. Regarding crosstalk between the HPA axis and glutamatergic signaling, activated GRs promoted glutamate efflux either by directly binding to presynaptic MRs [32, 33] or via indirect interaction with TrkB to potentiate BDNF‐evoked phospholipase C‐γ (PLC‐γ) signaling cascades [34] (Figure 1C1/2). Meanwhile, GR activation suppressed *BDNF* expression by directly binding to the early growth response protein 1 response element (EGR‐RE) located in the promoter region of *BDNF* exon IV [35]. Additionally, GRs increased hippocampal TrkB phosphorylation at the Y816 residue via its genomic action [36] (Figure 1D1/2). In turn, BDNF can induce GR phosphorylation at serine residues 155, 246, and 287 (S155/246/287) via intracellular Y705 and Y706 residues of TrkB [37–39] (Figure 1D3). The phosphorylated GRs then translocate into the nucleus to remodel the GC‐associated transcriptome, a regulatory cascade susceptible to perturbation by chronic stress or BDNF Val66Met polymorphism [37–39]. In terms of the crosstalk between GR signaling and neuroinflammation, pro‐inflammatory cytokines enhance HPA axis activity and promote GC secretion by targeting the hypothalamus, hippocampus, and PFC (Figure 1E1). In turn, activated GRs translocate into the nucleus to suppress pro‐inflammatory cytokine expression. Mechanistically, GRs directly interact with coactivators, including cAMP‐response element binding protein (CREB)‐binding protein (CBP) and p300/CBP‐associated factor (PCAF), thereby suppressing histone acetyltransferase activity and modulating lysine acetylation of core histone‐4 [40, 41] (Figure 1E2). Alternatively, activated GRs could indirectly recruit histone deacetylase (HDAC) and reverse histone acetylation to the nuclear factor kappa‐B (NF‐κB) complex [40, 41] (Figure 1E2). Moreover, activated GRs can translocate to mitochondria to modulate Ca^2+^ buffering capacity and reactive oxygen species (ROS) generation in an inverted U‐shaped dose‐dependent manner [42] (Figure 1F).

### 2.2. Monoamine Deficiency

Proposed over 60 years ago, the monoamine deficiency hypothesis has guided the development and clinical application of first‐line antidepressants, including tricyclic antidepressants (TCAs), monoamine oxidase inhibitors (MAOIs), and selective serotonin reuptake inhibitors (SSRIs). These pharmacological agents enhance synaptic/extracellular monoamine levels by suppressing monoamine degradation or transporter‐mediated reuptake, respectively [17]. Although monoamine‐targeted antidepressants can rapidly boost synaptic monoamine concentrations within hours, their behavioral therapeutic effects typically emerge after persistent treatment over 2 weeks. Accordingly, delayed transcriptional changes in genes encoding monoamine‐related receptors and transporters are regarded as essential mechanisms accounting for the delayed onset of antidepressant efficacy. In addition, conventional antidepressants often yield unsatisfactory outcomes against treatment‐resistant depression (TRD), a prevalent clinical phenotype affecting approximately one‐third of the depressed population [43]. Emerging evidence has revealed viable strategies for developing rapid‐onset, monoamine‐targeted antidepressants. Representative approaches encompass uncoupling the serotonin transporter (SERT) from nitric oxide synthase in the dorsal raphe nucleus (DRN) [44] and suppressing dopamine transporter activity [45]. Additionally, multiple novel regulatory mechanisms whereby non‐monoaminergic antidepressants target monoaminergic neurons have recently been identified, exemplified by ketamine‐mediated modulation of D1 dopamine receptors in the nucleus accumbens (NAc) [46]. Collectively, these findings indicate the feasibility of developing fast‐acting antidepressants with potent efficacy against TRD via targeted monoamine modulation. Meanwhile, monoamine‐independent therapeutic strategies have attracted growing attention and yielded novel progress, providing alternative avenues for optimized depression therapeutics [47, 48].

Among various monoamine neurotransmitters, serotonin (5‐HT) has garnered the most extensive research attention [17]. Over 90% systematic 5‐HT is synthesized in the intestines, which cannot penetrate the blood–brain barrier (BBB) to enter the brain parenchyma under physiological conditions. In intestinal tissues, its primary precursor tryptophan is converted to 5‐HT, catalyzed by tryptophan hydroxylase (TPH) 1 and aromatic amino acid decarboxylase (AAAD). Alternatively, tryptophan can be catabolized into kynurenine through the kynurenine pathway (KP), a process mediated by indoleamine 2,3‐dioxygenase (IDO) 1 and tryptophan‐2,3‐dioxygenase (TDO) [49, 50]. Unlike 5‐HT, tryptophan can cross the BBB and access the brain, where it undergoes two primary metabolic fates. Tryptophan can be metabolized to 5‐HT via the catalytic activity of TPH 2/AAAD or catabolized by IDO/TDO to generate kynurenine, which is further processed in a cell‐type‐specific manner [49, 50]. Specifically, kynurenine is converted into neurotoxic metabolite quinolinic acid (QUIN) via sequential enzymatic reactions catalyzed by kynurenine 3‐monooxygenase (KMO), kynureninase (KYNU), and 3‐hydroxyanthranilic acid oxygenase (3‐HAO) within microglia. Alternatively, it is transformed into neuroprotective kynurenic acid by kynurenine aminotransferases within astrocytes. Under pathological conditions (e.g., stress and inflammation), pivotal enzymes governing the KP (e.g., IDO and TDO) are markedly upregulated, and this metabolic shift promotes kynurenine production and suppresses 5‐HT biosynthesis. In line with this regulatory mechanism, rodent studies found that CUMS‐ or CRS‐triggered depressive‐like behavior was accompanied by increased kynurenine levels and diminished 5‐HT concentrations in the serum and hippocampus [51, 52]. These stress‐elicited metabolic and behavioral alterations were driven by enhanced IDO activity and suppressed TPH2 activity at both mRNA and protein levels, and such pathological abnormalities were effectively reversed by the IDO inhibitor 1‐MT [51, 52]. Intriguingly, 1‐week CRS reduced the hippocampal QUIN level while enhancing the kynurenic acid concentration in mice, whereas 4‐week CRS led to opposite effects, indicating a time‐dependent manner regarding stress‐modulated kynurenine metabolism [53, 54]. From an alternative perspective, probiotic intervention and tryptophan supplementation ameliorated depressive‐like phenotypes via normalizing chronic stress‐disrupted tryptophan metabolism [55, 56].

5‐HT receptors and transporters play pivotal roles in depression etiology [17, 57]. Among the over 14 identified 5‐HT receptor subtypes, the 5‐HT1A receptor has garnered the most attention. This receptor comprises presynaptic inhibitory autoreceptors and postsynaptic heteroreceptors, which are predominantly expressed in raphe nuclei and corticolimbic regions, mediating inhibitory and excitatory regulation of 5‐HT neuronal activity, respectively [58]. Presynaptic 5‐HT1A autoreceptors can suppress presynaptic 5‐HT release and modulate stress susceptibility and antidepressant responsiveness, given their specific expression within distinct subpopulations of serotonergic neurons [59, 60]. Meanwhile, growing evidence has highlighted the vital involvement of 5‐HT receptor polymorphisms, *SERT* gene polymorphisms, endocytic trafficking, and altered binding potential in depression pathogenesis [61–64]. In particular, the short allele of the SERT‐linked polymorphic region is tightly related to heightened stress susceptibility. Chronic stress can markedly remodel the expression and function of 5‐HT receptors and transporters and the overall activity of 5‐HT neuronal circuits [17, 57]. For instance, CUMS diminished the sensitivity of presynaptic autoreceptors, whereas MS increased the density and sensitivity of postsynaptic heteroreceptors across the dorsal raphe (DR), hippocampus, and amygdala in rats [65–67]. It is worth noting that 5‐HT neuronal activity in the DR exhibited distinct stress‐specific alterations: it was suppressed by CUMS whereas potentiated by chronic predator scent stress [65, 68]. Such divergent outcomes indicate that stress‐evoked serotonergic remodeling is contingent upon stress modality, wherein the CUMS and predator scent paradigms predominantly represent physical and psychological stressors, respectively.

### 2.3. Glutamate Excitotoxicity

#### 2.3.1. Glutamate Excitotoxicity in Depression

**Figure 2:**
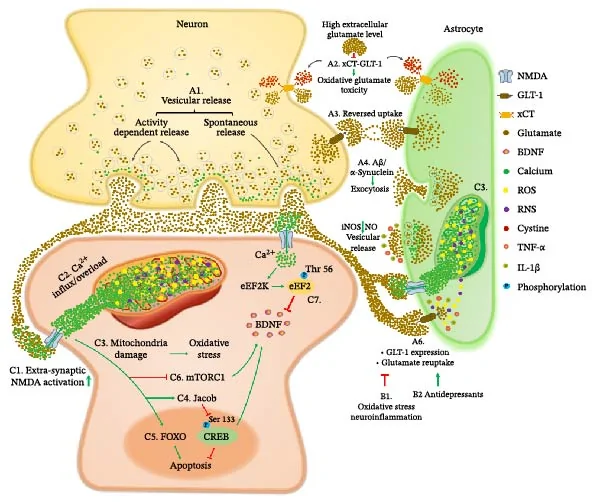
Involvement of glutamate excitotoxicity in the pathogenesis of depression. (A) Sources of extracullar glutamte include vesicular exocytosis from presynaptcic terminals (A1) or astrocytes (A4/A5), non‐vesicular efflux mediated by the xCT (A2) and reversed uptake (A3). (B) Disrupted astrocytic GLT‐1 reuptake triggers extracullar glutamate accumualtion and subsequent neuronal excitotoxicity. Oxidative stress and neuroinflammation (B1) impair extracullar glutamate clearance via suppressing the expression and functional activity of astrocytic GLT‐1 (A6), and this pathological alteration can be reversed by antidepressant interventions (B2). (C) Accelerated relasese and suppressed reuptake of glutamate synergistically exacerbate excessive extracullar glutamate accumualtion, ultimately triggering neuronal excitotoxicity. This pathological process is predominantly driven by extrasynaptic NMDA receptor overactivation, which induces sustained Ca^2+^ influx and intracellular overload, further leading to mitochondrial dysfunction and exaggerated oxidative stress (C1−3). Glutamate excitotoxicity ultimately promotes neuronal apoptosis via mutiple signaling cascades including Jacob and FOXO pathways (C4/5), and suppresses BDNF transcription via mTOR and eEF2 signaling (C6/7). BDNF, brain‐derived neurotrophic factor; CREB, cAMP‐response element binding protein; FOXO, forkhead box O; GLT‐1, glutamate transporter 1; IL‐1β, interleukin‐1β; iNOS, inducible nitric oxide synthase; Jacob, juxta synaptic attractor of caldendrin on dendritic boutons protein; mTOR, mammalian target of rapamycin; NMDA, N‐methyl‐D‐aspartic acid; NO, nitric oxide; RNS, reactive nitrogen species; ROS, reactive oxygen species; TNF‐α, tumor necrosis factor‐α; xCT/System Xc‐, cystine/glutamate antiporter system.

Glutamate serves as the predominant excitatory neurotransmitter in the brain, occupying ~80% of cortical synapses and being synthesized de novo from α‐ketoglutarate in neurons and astrocytes [69, 70]. Synaptic glutamate primarily derives from vesicular exocytosis at presynaptic terminals, in a Ca^2+^ and vesicular glutamate transporter (GLT)‐dependent manner, encompassing both spontaneous release and neuronal activity‐triggered release [71–73] (Figure 2A1). These two release modes differ in kinetic properties and selectively activate distinct postsynaptic NMDA receptor subtypes [71–73]. Astrocytes also contribute to extracellular glutamate via non‐vesicular release, including efflux mediated by the cystine‐glutamate antiporter (system Xc‐/xCT) and reversed transporter uptake [74, 75] (Figure 2A2/3), α‐Synuclein or amyloid‐β peptide‐stimulated exocytosis [76, 77] (Figure 2A4), and neuroinflammation‐promoted vesicular release [78, 79] (Figure 2A5). There exists a striking concentration gradient of glutamate across brain cellular compartments. Intracellular glutamate concentration reaches ~10 mmol/L in the neuronal cytoplasm and 100 mmol/L within vesicles, whereas astrocytic glutamate is maintained at around 0.3 mmol/L, all substantially higher than extracellular glutamate levels (1–30 μmol/L) [80, 81]. Furthermore, neuronal activation triggers a transient synaptic glutamate increase, surging from a basal level of ~20 to 1–3 mmol/L, driven by robust vesicular glutamate exocytosis [80, 81].

Excessive glutamate release or interrupted glutamate reuptake elicits neuronal excitotoxicity, a pathogenic mechanism initially proposed nearly four decades ago [82]. Glutamate excitotoxicity is predominantly driven by the overactivation of extrasynaptic ionotropic N‐methyl‐D‐aspartic acid (NMDA) receptors, which triggers sustained Ca^2+^ influx and subsequent intracellular overload, eventually causing mitochondrial damage [83, 84] (Figure 2C1–3). This process further activates a series of cell death‐associated signaling cascades and regulatory molecules, such as forkhead box O and Jacob proteins, which collectively drive apoptosis and necrosis [83, 84] (Figure 2C4−5). Cumulative evidence underscores the crucial involvement of central glutamate excitotoxicity in depression pathophysiology, highlighting glutamatergic modulation as a promising therapeutic avenue [18, 85–87]. For example, an early postmortem study detected an increased glutamate concentration in the PFC of depressed individuals [88]. Subsequent proton magnetic resonance spectroscopy (^1^H‐MRS) studies have reported inconsistent glutamatergic alterations owing to confounding variables including depression subtypes, disease stages, and specific brain subregions [89, 90]. Notably, ^1^H‐MRS possesses inherent technical limitations: this technique cannot distinguish glutamate from glutamine, nor can it discriminate intracellular glutamate from extracellular counterparts or synaptic pools from extrasynaptic ones. In line with clinical observations, rodent studies found that stress or corticosterone elicited rapid and sustained glutamate efflux in the hippocampus, PFC, and amygdala, respectively [91–93]. CUMS and CRS exacerbated methamphetamine‐evoked glutamate excitotoxicity in the rat striatum [94, 95]. Furthermore, CUMS potentiated glutamatergic transmission in the PFC, while CRS enhanced glutamate neuronal activity within medial preoptic area circuits in mice [96, 97]. Suppression of such hyperactive glutamatergic signaling effectively mitigated depressive‐like behavior [97]. In addition, CUMS increased the hippocampal glutamate level, as quantified via biochemical assays, and such pathological perturbations were reversed by fluoxetine or Xiaoyao San [98, 99]. Nevertheless, the acute and long‐term in vivo effects of chronic stress on glutamatergic dynamics remain poorly elucidated. This knowledge gap largely stems from the high lability of extracellular glutamate, which is tightly modulated by activity‐dependent rapid neuronal exocytosis, astrocytic reuptake, and multifaceted neural adaptive responses induced by persistent stress. Advanced neurotransmitter monitoring techniques featuring high spatiotemporal resolution, such as fluorescent probes, hold great promise for unraveling the mystery.

Glutamate cannot cross the BBB into peripheral circulation, whereas the synaptic cleft lacks glutamate‐degrading enzymes. Accordingly, reuptake serves as the predominant terminating mechanism of synaptic glutamatergic signaling, and impairment of this process would trigger extracellular glutamate accumulation and subsequent neuronal excitotoxicity [100] (Figure 2A6). Extracellular glutamate is predominantly internalized via GLT‐1, which is mainly expressed in astrocytes [101, 102]. Approximately 5%–10% of GLT‐1 is expressed at neuronal axon terminals in the hippocampus and cortex, devoted to mitochondrial metabolism [103, 104]. *GLT-1* mutant mice exhibited a glutamate reuptake efficiency as low as 5.8% relative to wild‐type counterparts, indicating astrocytic GLT‐1 as the protagonist responsible for reuptake over 90% of extracellular glutamate [105]. Mounting evidence demonstrated that CUMS‐evoked depressive‐like behavior was tightly linked to downregulation and functional suppression of astrocytic GLT‐1 in the rodent hippocampus and PFC [106, 107] (Figure 2B1). Consistently, targeted GLT‐1 inhibition was sufficient to induce robust depressive‐like phenotypes in rodents, as evidenced by microinjection of the selective antagonist dihydrokainic acid (DHK) into multiple brain subregions (e.g., lateral ventricle, PFC, amygdala, and habenula) [108–111], *GLT-1* knockout or knockdown [103, 112–115], and chronic stress exposure [15, 106]. In contrast, genetic overexpression or pharmacological activation of GLT‐1, induced by agents such as ceftriaxone and riluzole, exerted robust antidepressant effects [116–119]. Moreover, antidepressants (e.g., fluoxetine and ketamine) or antidepressant compounds (e.g., the Chinese herbal formula Xiaoyao San) effectively rescued depressive‐like behavior, elicited by chronic stress‐inhibited GLT‐1 function [15, 106] and *GLT-1* knockdown [115]. Collectively, these findings highlight the essential role of central astrocytic GLT‐1 in depression pathogenesis and therapeutic intervention, respectively [120].

Beyond GLT‐1 modulation, alterations in the expression and function of the glutamate receptor (GluR) are strongly implicated in depression pathophysiology, which can be effectively reversed by antidepressants and antidepressant compounds [85]. Accumulating preclinical and clinical evidence underscores the dual regulatory role of NMDA modulation in depression: NMDA inhibition exerts robust antidepressant efficacy, whereas NMDA activation is concurrently essential for the therapeutic benefits of antidepressants [18, 121]. On the one hand, a precious postmortem study demonstrated that the expression of NR2A and NR2B subunits was reduced by ~50% in the PFC and olfactory cortex of depressed individuals [122]. Consistently, CUMS markedly suppressed neuronal NR2B expression in the rodent hippocampus and anterior marginal cortex, reversed by the antidepressant vortioxetine or Xiaoyao San [98, 123]. On the other hand, multiple NMDA antagonists (e.g., ketamine, MK‐801, and CPP) exerted robust antidepressant actions [124, 125]. Notably, ketamine specifically blocked NMDA receptor activity in neurons of the lateral habenula (LHb) rather than the hippocampus in mice, revealing a brain region‐specific mechanism [126]. Furthermore, ketamine exerted sustained antidepressant action by blocking extrasynaptic NMDA receptors and NMDA receptors expressed on GABAergic inhibitory interneurons or via NMDA trapping within the LHb [127, 128]. In contrast, MK‐801 or CPP pretreatment abolished the antidepressant efficacy of ketamine, substantiating the essential role of NMDA receptor activation in ketamine‐mediated antidepressant action [129]. These paradoxical findings indicate that both activation and suppression of NMDA receptors are critically involved in the therapeutic actions of ketamine. Furthermore, compelling preclinical evidence suggests that synaptic and extrasynaptic NMDA receptors mediate distinct, even antagonistic, biological functions [83, 130]. Specifically, these two receptor subtypes differentially modulate diverse cellular processes, such as neuronal Ca^2+^ signaling, CREB activity, and *BDNF* expression, thereby selectively promoting neuronal survival or apoptotic cell death, respectively. These findings indicate lingering uncertainties regarding NMDA receptor‐mediated regulatory patterns, including dichotomous effects of NMDA activation versus inhibition, functional discrepancies between synaptic and extrasynaptic NMDA receptors, and functional heterogeneity across distinct brain subregions. Despite remaining inconsistencies, these findings at least solidify the essential role of NMDA receptors in depression pathophysiology, thereby rationalizing NMDA modulation as a promising therapeutic strategy [131]. Moreover, (2R,6R)‐hydroxynorketamine, a major bioactive metabolite of ketamine, exerted potent antidepressant action via modulating metabotropic GluR (mGluR) 2 rather than NMDA receptors, emphasizing the crucial contribution of mGluR signaling to antidepressant responses [132, 133]. The vital involvement of mGluRs and AMPA receptors in chronic stress‐induced depressive phenotypes has been previously elucidated [134, 135].

#### 2.3.2. Reciprocal Crosstalk Between Glutamate Excitotoxicity and Other Pathogenic Mechanisms

Glutamate excitotoxicity reciprocally interacts with multiple pathogenic cascades involved in depression, as illustrated in Figure 2. Excessive extracellular glutamate induces oxidative stress via over‐activating extrasynaptic NMDA receptors and causing mitochondrial damage [21, 83] (Figure 2C1−3) while concurrently suppressing astrocytic GLT‐1 function or disrupting cooperation between xCT and GLT‐1 [136–138]. In turn, excessive ROS accelerate extracellular glutamate accumulation by facilitating spontaneous vesicular release and reversed uptake while suppressing GLT‐1 activity and compromising astrocytic reuptake capacity [139–142]. Regarding glutamate and BDNF interplay, excessive extracellular glutamate suppresses BDNF synthesis via over‐activating extrasynaptic NMDA, which subsequently inhibits phosphorylation of mammalian target of rapamycin (mTOR) or phosphorylation of CREB at serine 133 through Jacob signaling [83, 130] (Figure 2C4/6). Alternatively, this inhibitory effect may be mediated by eukaryotic elongation factor 2 (eEF2) signaling, given that spontaneous synaptic NMDA blockade exerted antidepressant action via suppressing eEF2 phosphorylation at threonine 56, which ultimately enhanced *BDNF* expression [125, 127, 143] (Figure 2C7). In turn, BNDF can modulate presynaptic glutamate release and regulates the expression, subcellular localizations, and activation of postsynaptic GluRs [144]. Paradoxically, BDNF protected neurons against glutamate excitotoxicity, through ERK and PI3‐kinase signaling [145] and modulating intracellular Ca^2+^ homeostasis and inflammatory cytokine expression [146, 147]. Additionally, excessive glutamate promoted neuroinflammation via stimulating α‐amino‐3‐hydroxy‐5‐methyl‐4‐isoxazolepropionic (AMPA) receptors, thereby promoting microglial secretion of tumor necrosis factor‐α (TNF‐α) and interleukin‐1β (IL‐1β) [148, 149]. In a positive feedback loop, pro‐inflammatory cytokines further exacerbate glutamatergic dysregulation. They impair astrocytic glutamate reuptake [150] and promote glutamate release from microglia and astrocytes, mediated by increased glutaminase activity modulated by protein kinase C and CREB [151, 152], and nitric oxide synthase and nitric oxide signaling [78, 79].

### 2.4. BDNF/TrkB Dysregulation

BDNF is a major type of neurotrophic factor secreted by a wide range of cell types and crucially involved in neuronal plasticity modulation [153]. Compelling preclinical and clinical evidence underscores the critical role of central BDNF/TrkB signaling in depression pathogenesis and the pharmacological efficacy of antidepressant regimens [19, 154]. Unlike the consistent alterations in synaptic 5‐HT depletion and hyperactive glutamatergic signaling, depression‐associated BDNF/TrkB dysregulation seems to exhibit a highly heterogeneous, brain‐region‐dependent expression pattern. Existing evidence indicates BDNF/TrkB signaling is generally suppressed in the hippocampus and PFC whereas potentiated within the amygdala and NAc [19]. Clinically, postmortem brain analyses revealed markedly decreased BDNF mRNA and protein levels in the hippocampus and PFC but increased *BDNF* expression in the NAc and amygdala in depressed patients [153, 155, 156].

Preclinical rodent models provide primary evidence illustrating the bidirectional, region‐dependent regulatory properties of BDNF/TrkB signaling implicated in depression [19, 154]. Specifically, disruption and potentiation of BDNF/TrkB signaling in the hippocampus and PFC exert potent pro‐depressant and anti‐depressant effects, respectively. First, BDNF signaling inhibition in these two regions was closely associated with the emergence of depressive‐like phenotypes elicited by CUMS or CSDS, whereas microglial BDNF depletion markedly increased the susceptibility of mice and rats to CUMS‐elicited depressive‐like behavior [157–161]. Furthermore, exogenous BDNF delivery, either through peripheral mini‐pump infusion or quercetin‐based alginate nanogels delivery, effectively alleviated CUMS‐induced depressive‐like behavior [162, 163]. Additionally, accumulating evidence indicates that intact BDNF/TrkB signaling serves as an essential prerequisite for the therapeutic efficacy of diverse antidepressant agents. This mechanism underpins the actions of scopolamine and (2R,6R)‐hydroxynorketamine in *BDNF* Val66Met knock‐in mice [164, 165] and in mice subjected to CUMS or CRS [166, 167]. Consistently, genetic ablation or pharmacological inhibition of BDNF signaling is sufficient to abrogate antidepressant therapeutic benefits. Specifically, microinjection of neutralizing BDNF antibody into the medial PFC (mPFC) [168] and *BDNF* Val‐to‐Met mutation or targeted *BDNF* gene knockout [125, 169] effectively blocked ketamine‐elicited antidepressant responses in mice. In line with these findings, heterozygous BDNF‐null (BDNF^+/−^) mice exhibited behavioral resistance to conventional antidepressants, including imipramine and fluoxetine [170]. However, central *BDNF* expression was significantly upregulated by venlafaxine and sertraline but not affected by escitalopram [171, 172]. These results might suggest that enhanced *BDNF* expression severs as a sufficient yet non‐essential modulator for triggering antidepressant responses. Regarding the molecular mechanism underlying antidepressant‐induced BDNF modulation, arketamine exerted potent antidepressant action in CSDS‐susceptible mice via promoting microglial BDNF synthesis, mediated by phosphorylation of CREB at the S133 residue and methyl‐CpG‐binding protein 2 (MeCP2) at the S421 residue [173]. Moreover, BDNF‐dependent MeCP2 phosphorylation was proposed to be exclusively implicated in mediating the sustained, rather than rapid, antidepressant effects of ketamine and scopolamine [174].

Apart from BDNF‐dependent modulation, growing evidence has identified the antidepressant efficacy of direct TrkB activation by both monoamine‐targeted and glutamate‐modulating antidepressants. Multiple representative antidepressants, including fluoxetine, imipramine, and ketamine, directly bound to and activated TrkB receptors in cortical neurons, which promoted TrkB phosphorylation at the PLC‐γ1‐interacting Y816 residue, thereby facilitating TrkB synaptic localization and activation [175]. Furthermore, site‐directed mutagenesis of the TrkB receptor, in which the tyrosine residue at the Y433 locus was substituted with phenylalanine, compromised their antidepressant efficacy [175]. In addition, mice overexpressing the TrkB.T1 isoform exhibited prominent resistance to imipramine and fluoxetine treatment [170]. In parallel, pharmacological suppression of endogenous TrkB activity by the tyrosine kinase antagonist K252a was sufficient to abolish the antidepressant efficacy of ketamine in CUMS‐treated rats [176]. Collectively, these findings indicate that functional TrkB activation might serve as an essential prerequisite for mediating antidepressant responses. Notably, TrkB‐dependent antidepressant mechanisms seem to exhibit regional heterogeneity across the brain, consistent with region‐specific BDNF alterations. Specifically, local microinjection of the TrkB agonist 7,8‐dihydroxyflavone into the hippocampus and PFC or infusion of the TrkB antagonist ANA‐12 into the NAc elicited potent antidepressant action in rats and mice, respectively [177, 178]. To date, whether subregion‐specific discrepancies in TrkB activation patterns drive heterogeneous therapeutic responses to distinct antidepressant agents remains poorly deciphered.

Dysregulated BDNF/TrkB signaling reciprocally interacts with multiple pathogenic processes underlying depression, especially HPA axis dysregulation and glutamate excitotoxicity. The crosstalk between BDNF/TrkB signaling and the HPA axis primarily relies on the direct functional interplay between TrkB receptors and GRs. Mechanistically, BDNF signaling dose‐dependently rewrote the GC transcriptome via phosphorylating GR at serine residues 155, 246, and 287 (S155/246/287) [38, 39] (Figure 1D3), and this regulatory process was disrupted by the BDNF Val66Met polymorphism [37]. In turn, activated GRs exert bidirectional modulation of BDNF/TrkB signaling: on the one hand, GRs promoted hippocampal TrkB phosphorylation at the Y816 residue via canonical genomic pathways [36] (Figure 1D1); on the other hand, activated GRs suppressed BDNF transcription via directly binding to the EGR‐RE motif, located in the promoter region of BDNF exon IV [35] (Figure 1D2). In terms of glutamatergic crosstalk, BDNF bidirectionally modulates presynaptic glutamate release and postsynaptic receptor activity [144]. Mechanistically, BDNF‐triggered TrkB activation facilitated rapid glutamate efflux via the downstream PLC‐γ/IP3/Ca^2+^ and Src/PLC‐γ1 signaling cascades, and this pro‐glutamatergic effect was further potentiated by antidepressants, whereas fully abrogated by the TrkB antagonist K252a [179–182]. Paradoxically, BDNF protected neurons against glutamate excitotoxicity through ERK and PI3‐kinase signaling and modulating intracellular Ca^2+^ homeostasis and inflammatory cytokine expression [145–147]. Such bidirectional regulatory effects suggest that the biological outcomes of BDNF signaling are highly context‐dependent, relying on the basal physiological status and ambient glutamate concentrations within the neural microenvironment. Reciprocally, excessive extracellular glutamate suppresses BDNF synthesis via extrasynaptic NMDA receptor‐initiated mTOR and CREB signaling inhibition (Figure 2C4/6) or presumably via synaptic NMDA receptor‐mediated eEF2 signaling [83] (Figure 2C7), as mentioned above. To date, the precise physiological and pathological conditions governing the selective activation of these distinct regulatory pathways remain poorly defined. Additionally, BDNF modulates neuroinflammation via TrkB activation that boosts NF‐κB activity and upregulates pro‐inflammatory cytokine expression, which subsequently promote *BDNF* expression in a feedback manner whose underlying regulatory mechanisms remain uncharacterized [183].

### 2.5. Neuroinflammation

#### 2.5.1. Neuroinflammation in Depression

**Figure 3:**
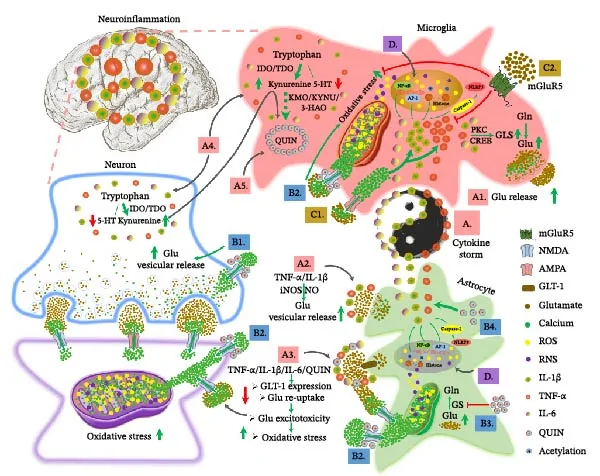
Involvement of neuroinflammation in the pathogenesis of depression. (A) Excessive pro‐inflammatory cytokines mainly secreted by activated cerebral microglia trigger a cytokine storm under acute pathological conditions. Neuroinflammation drives glutamate excitotoxicity via multiple regulatory mechanisms: it augments microglial glutamate release via enhancing GLS activity (A1), facilitates astrocytic vesicular secretion via the iNOS and NO signaling (A2), and inhibits astrocytic GLT‐1 expression and glutamate reuptake (A3). Additionally, neuroinflammation promotes kynurenine pathway of tryptophan metabolism by upregulating IDO and TDO enzymatic activities, thereby suppressing 5‐HT biosynthesis and increasing kynurenine production (A4). Accumulated kynurenine is further metabolized to QUIN, via KMO, KYNU, and 3‐HAO in brain microglia (A5). (B) QUIN serves as an NMDA agonist and exacerbates depressive pathological processes via multiple pathways: it promotes vesicular glutamate release (B1), potentiate oxidative stress (B2), suppress GS activity (B3), and elevates pro‐inflammatory cytokine expression (B4). (C) Glutamate exerts bidirectional modulation on microglial inflammation: it enhances TNF‐α and IL‐1β release via AMPA receptor activation (C1), while suppressing TNF‐α and ROS generation through mGluR5 signaling (C2). (D) Excessive ROS further promotes pro‐inflammatory cytokine gene transcription by activating AP‐1 and NF‐κB and accelerating histone acetylation modification. 3‐HAO, 3‐hydroxyanthranilic acid oxygenase; 5‐HT, serotonin; AMPA, α‐aino‐3‐hydroxmy‐5‐methyl‐4‐isoxazole‐propionic acid; AP‐1, activating protein‐1; CREB, cAMP responsive element binding protein; Gln, glutamine; GLS, glutaminase; GLT‐1, glutamate transporter 1; Glu, glutamate; GS, glutamine synthetase; IDO, indoleamine 2,3‐dioxygenase; IL‐1β, interleukin‐1β; IL‐6, interleukin‐6; iNOS, inducible nitric oxide synthase; KMO, kynurenine 3‐monooxygenase; KYNU, kynureninase; NF‐κB, nuclear factor kappa‐B; NLRP3, NOD‐like receptor thermal protein domain associated protein 3; NMDA, N‐methyl‐D‐aspartic acid; NO, nitric oxide; PKC, protein kinase C; QUIN, quinolinic acid; RNS, reactive nitrogen species; ROS, reactive oxygen species; TDO, tryptophan‐2,3‐dioxygenase; TNF‐α, tumor necrosis factor‐α.

Neuroinflammation refers to the immune response of neurons and glial cells (predominantly microglia) to disrupted cerebral homeostasis. The process is characterized by robust activation of immune‐related transcription factors and increased expression of inflammatory mediators, such as NF‐κB, IL‐1β, interleukin‐6 (IL‐6), TNF‐α, inflammasome NOD‐like receptor thermal protein domain‐associated protein 3 (NLRP3), and ROS [184, 185]. The central nervous system maintains extensive reciprocal crosstalk with peripheral circulation via immune responses, blood circulation, and neural networks. Peripheral pro‐inflammatory cytokines (e.g., IL‐1β, IL‐6, and TNF‐α), which are markedly upregulated upon LPS challenge, can infiltrate the brain and trigger neuroinflammatory cascades. Notably, the LPS‐induced rodent model has been widely adopted in depression research, given its potent pro‐inflammatory effects and high efficiency in eliciting depressive‐like phenotypes. This peripheral‐to‐central inflammatory transmission occurs mainly via BBB disruption [186, 187], while emerging evidence has uncovered previously unrecognized pathways, such as the recently characterized velum interpositum route [188]. Excessive pro‐inflammatory cytokines trigger an intracerebral cytokine storm (Figure 3A), a core pathological mechanism that drives depressive‐like behavior in the LPS‐induced model and accounts for increased incidence of depression during the COVID‐19 epidemic [189–191].

Neuroinflammation is critically implicated in the pathogenesis of numerous neurological disorders [192]. Accumulating evidence has identified its vital involvement in depression, initially inspired by the clinical observation that hepatitis C patients following interferon‐α (IFN‐α) therapy frequently develop severe depressive symptoms [193, 194]. Clinical investigations detected markedly increased levels of pro‐inflammatory cytokines, such as IL‐1β, IL‐6, and TNF‐α, alongside the neuroinflammation biomarker 18‐kDa translocator protein, in peripheral serum, CSF, and key brain subregions (e.g., hippocampus and PFC) in depressed individuals, reversed by antidepressant treatments [195–197]. Consistently, preclinical rodent studies further corroborate the close association between stress‐elicited neuroinflammation and depressive‐like phenotypes. Multiple chronic stress paradigms, such as MS and CUMS, markedly upregulated the expression of IL‐1β, IL‐6, TNF‐α, INF‐γ, NLRP3, and NF‐κB within the rodent hippocampus and PFC [198–201]. Increasing evidence highlights NLRP3 inflammasome activation as a pivotal inflammatory driver of depression. For instance, hippocampal NLRP3 overexpression elicited depressive‐like behavior in mice [202], whereas genetic suppression of NLRP3, or fecal microbiota transplantation (FMT) from NLRP3‐knockout mice, effectively alleviated depressive‐like phenotypes [202, 203]. These observations indicate that bidirectional modulation of NLRP3 signaling contributes substantially to the initiation and remission of depression, respectively.

Growing evidence has revealed antidepressant efficacy of anti‐inflammatory compounds [204] and anti‐inflammatory properties of antidepressants (e.g., SSRIs and fluoxetine) [196, 197]. These observations strongly support targeting neuroinflammation as a novel therapeutic direction for developing precision antidepressant strategies [205, 206]. On the one hand, multiple anti‐inflammatory agents, including berberine, geraniol, limonene, and progesterone, effectively ameliorated depressive‐like phenotypes in rodent models [207–210]. Mechanistically, these compounds normalized the upregulation of pro‐inflammatory mediators (e.g., IL‐1β, TNF‐α, and NLRP3) caused by MS, CUMS, or corticosterone treatment [207–210]. On the other hand, fluoxetine effectively normalized the upregulation of IL‐1β, IL‐6, and TNF‐α, in the hippocampus and PFC of rodents subjected to CUMS exposure or LPS challenge [211, 212]. Collectively, these findings further endorse the proposition that immunomodulation may serve as a viable therapeutic strategy [186, 213]. Notably, the acute LPS‐induced rodent model provides compelling evidence highlighting the pivotal role of neuroinflammation in depression pathogenesis [214, 215]. In comparison, the mechanistic linkage between neuroinflammation and depressive phenotypes remains poorly characterized in chronic stress paradigms. This research gap can be partially attributable to the mild pro‐inflammatory potency triggered by chronic stress, which is concurrently counterbalanced by sustained endogenous anti‐inflammatory compensatory responses throughout prolonged stress exposure.

#### 2.5.2. Reciprocal Crosstalk Between Neuroinflammation and Other Pathogenic Mechanisms

Neuroinflammation engages in intricate crosstalk with multiple depression‐associated pathological processes, as illustrated in Figure 3. Regarding its interplay with the HPA axis, clinical studies found a robust positive correlation between elevated IL‐6 levels and HPA axis hyperactivity, alongside a negative association between IL‐1β mRNA expression and morning cortisol reactivity [216, 217]. Pro‐inflammatory cytokines drive excessive HPA axis activation and augment GC secretion via directly targeting the hypothalamus or indirectly modulating hippocampal and PFC function [41, 218] (Figure 1E1). In turn, GC‐activated GRs translocate into the nucleus to suppress pro‐inflammatory cytokine expression via directly binding to coactivators (e.g., CBP and PCAF) or indirectly recruiting HDACs (Figure 1E2). Synthetic GCs have been clinically applied for the treatment of immune‐related disorders since 1948, whose effect, however, is pleiotropic and context‐dependent, modulated by multiple variables including drug dosage and administration timing. Specifically, high‐dose GCs suppressed pro‐inflammatory cytokine expression, whereas low‐dose GCs exerted the opposite facilitatory effect [219]. Furthermore, GC pretreatment prior to LPS exposure potentiated inflammatory responses, whereas GC administration following the LPS challenge effectively attenuated cytokine expression [220].

Inflammatory activation could inhibit 5‐HT generation through enhancing IDO/TDO activity and facilitating the production of kynurenine, which is then metabolized into QUIN in microglia of the brain, catalyzed by KMO, KYNU, and 3‐HAO [49, 193, 221] (Figure 3A4/5). Clinical and preclinical evidence corroborates this regulatory cascade: IFN‐α promoted IDO activity and enhanced cerebral kynurenine and QUIN levels in clinical cohorts [222], whereas CUMS and CRS triggered 5‐HT depletion and exacerbated kynurenine accumulation in the serum and hippocampus of mice via facilitating IDO/TDO activity [51, 55]. It is worth noting that QUIN cannot cross the BBB, while the brain lacks an efficient reuptake mechanism to clear extracellular QUIN. As a result, sustained QUIN production drives its extracellular accumulation and further triggers intracerebral neurotoxicity. Excessive QUIN drives glutamate excitotoxicity via inhibiting astrocytic reuptake, (Figure 3A3), stimulating vesicular glutamate release (Figure 3B1), or suppressing glutamine synthetase activity (Figure 3B3) [223–225] (Figure 3A3/B1/B3). Acting as a potent NMDA agonist, excessive QUIN also overstimulates NMDA receptors, thereby provoking sustained Ca^2+^ influx and intracellular overload and subsequently resulting in mitochondrial damage and robust oxidative stress [223, 224, 226] (Figure 3B2). Furthermore, QUIN may further amplify neuroinflammatory responses indirectly through excessive oxidative stress, which promotes histone acetylation and subsequently activates protein‐1 (AP‐1) and NF‐κB signaling cascades [227, 228] (Figure 3B4/D). Additionally, antidepressant treatment effectively suppressed inflammation‐induced QUIN overproduction, further validating the crucial involvement of aberrant QUIN metabolism in depression pathophysiology from another perspective [222, 229]. Nevertheless, the detailed molecular mechanisms underlying the multifaceted neurobiological actions of QUIN remain to be further elucidated.

The reciprocal crosstalk between neuroinflammation and glutamatergic signaling is critically implicated in depression [230, 231]. Clinically, IFN‐α administration increased glutamate concentration in the basal ganglia and dorsal anterior cingulate cortex of depressed patients [232, 233]. Neuroinflammation‐driven increase in the extracellular glutamate level is predominantly ascribed to aberrant glutamate efflux from activated microglia and astrocytes. Multiple preclinical studies provided compelling supportive evidence: LPS challenge promoted microglial glutamate release in the rodent hippocampus [234, 235], whereas IL‐1β and TNF‐α facilitated astrocytic vesicular glutamate secretion [78, 79] (Figures 2A5 and 3A2). Paradoxically, activated microglia also abundantly expressed GLT‐1, which can promote extracellular glutamate reuptake [236–238]. Furthermore, this regulatory effect appears to be inflammation‐dependent, as LPS‐induced microglial GLT‐1 upregulation relied on concurrent TNF‐α release and was abrogated by the anti‐inflammatory corticosterone [239, 240]. The concurrent elevations of extracellular glutamate and microglial GLT‐1 expression constitute an apparent paradox. This discrepancy can be partially explained by in vitro evidence demonstrating that LPS exposure markedly inhibited the expression of astrocytic GLT‐1 and glutamate‐aspartate transporter in mixed microglia‐astrocyte cultures, thereby impairing astrocytic glutamate reuptake capacity—the primary mechanism for extracellular glutamate clearance [150, 241] (Figures 2A6 and 3A3). Collectively, neuroinflammation imposes complex and context‐dependent modulation on glutamatergic homeostasis. This process is shaped by both intrinsic and extrinsic factors that govern glutamate release and reuptake, encompassing macroenvironment (e.g., physiological and pathological states) and microenvironment cellular composition (e.g., microglia and astrocytes). Reciprocally, glutamatergic signaling bidirectionally regulates neuroinflammation via distinct microglial GluRs. Glutamate may drive neuroinflammation by acting on microglial AMPA and NMDA receptors, whereas it attenuates inflammatory activity via mGluR5 signaling. As evidence, glutamate amplified neuroinflammatory responses by facilitating TNF‐α and IL‐1β release via AMPA receptors in cultured rat microglia [148, 149] (Figure 3C1). Furthermore, pharmacological blockade of NMDA receptors by MK801 mitigated the hypoxia‐induced release of TNF‐α and IL‐1β in cultured microglia [242]. In contrast, mGluR5 activation suppressed LPS‐promoted microglial release of TNF‐α and ROS, which was abrogated in mGluR5‐knockout mice or blocked by mGluR5 antagonist treatment, suggesting the putative anti‐inflammatory property of mGluR5 activation [243, 244] (Figure 3C2).

### 2.6. Oxidative Stress

#### 2.6.1. Oxidative Stress in Depression

**Figure 4:**
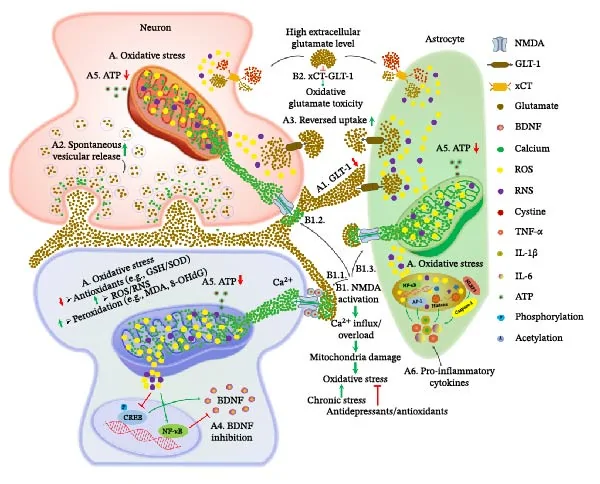
Involvement of central oxidative stress in the pathogenesis of depression. (A) Excessive oxidative stress exerts multifaceted pathological effects: it drives glutamate excitotoxicity by suppressing astrocytic GLT‐1 expression and activity, promoting spontaneous vesicular glutamate release, and accelerating reversed glutamate uptake (A1−3); it inhibits *BDNF* expression by diminishing CREB activity and potentiating NF‐κB DNA binding affinity (A4), triggers intracellular ATP depletion (A5), and promotes neuroinflammation via AP‐1/NF‐κB signaling and enhanced histone acetylation (A6). In turn, excessive extracellular glutamate and QUIN further aggravates oxidative stress via two major pathways: they overactivate extrasynaptic NMDA receptors to trigger sustained Ca^2+^ influx and intracellular overload, which impairs mitochondrial function and boosts ROS/RNS generation (B1.1–1.3); they disrupt the functional cooperation between astrocytic GLT‐1 and xCT, ultimately inducing oxidative glutamate toxicity (B2). 8‐OHdG, 8‐hydroxydeoxyguanosine; AP‐1, activating protein‐1; ATP, adenosine triphosphate; BDNF, brain‐derived neurotrophic factor; CREB, cAMP responsive element‐binding protein; GLT‐1, glutamate transporter 1; GSH, glutathione; MDA, malondialdehyde; NF‐κB, nuclear factor kappa‐B; NMDA, N‐methyl‐D‐aspartic acid; RNS, reactive nitrogen species; ROS, reactive oxygen species; SOD, superoxide dismutase; xCT/System Xc‐, cystine/glutamate antiporter system.

Oxidation is an essential cellular redox reaction that generates free radicals, such as ROS and reactive nitrogen species, which serve as pivotal signaling mediators under physiological conditions. Antioxidant enzymes and small molecules, such as superoxide dismutase (SOD) and glutathione (GSH), maintain redox homeostasis and defend cells against lipid peroxidation. Excessive oxidative activity or compromised antioxidant capacity triggers peroxidative damage to nucleic acids, lipids, and proteins, ultimately causing cellular injury—a pathological state commonly referred to as oxidative stress [245] (Figure 4A). As major endogenous sources of ROS, the mitochondrial respiratory chain and NADPH oxidase (NOX) are abundantly expressed in brain neurons, astrocytes, and microglia. This feature endows the brain with robust ROS production capability. Nevertheless, the brain is inherently highly susceptible to exaggerated oxidative stress owing to innate properties of high oxygen consumption, abundant unsaturated fatty acids, and relatively limited antioxidant capacity [246].

Cumulative evidence highlights the pivotal involvement of oxidative stress in depression pathogenesis, thereby rationalizing antioxidant‐targeted therapy as a promising therapeutic option [21, 247]. Clinically, multiple studies found reduced SOD activity and increased levels of malondialdehyde (MDA) and 8‐hydroxydeoxyguanosine in the serum of depressed individuals [248, 249]. Consistently, rodent studies demonstrated chronic stress paradigms (e.g., MS, CUMS, and CRS) markedly suppressed the activity of antioxidant molecules (e.g., GSH and SOD), while boosting expression of ROS and peroxidative makers (e.g., MDA, 4‐hydroxynonenal, and carbonyl), in the hippocampus and PFC, respectively [15, 198, 209, 250, 251]. Furthermore, accumulating evidence corroborates a reciprocal regulatory relationship between antioxidants and antidepressant efficacy, further validating the causal link from another perspective. Specifically, conventional antidepressants possess potent antioxidant properties, whereas multiple antioxidants exert robust antidepressant actions. Clinically, fluoxetine and citalopram enhanced SOD activity, whereas reduced peripheral MDA and carbonyl levels in depressed individuals [252, 253]. In preclinical studies, fluoxetine and agomelatine effectively alleviated CSDS‐ or MSSI‐elicited depressive‐like behavior in rodents by normalizing stress‐induced downregulation of SOD/GSH and upregulation of MDA/4‐HEN within the hippocampus [15, 254]. Complementarily, pharmacological interventions targeting oxidative stress ameliorate stress‐elicited depressive‐like phenotypes. Multiple natural and synthetic antioxidants (e.g., N‐acetylcysteine and diallyl disulfide) [15, 255, 256] and antioxidant‐active compounds (e.g., anethole and limonene) [208, 251] effectively alleviated depressive‐like behavior elicited by chronic stress (e.g., MS, CUMS, and CRS) in rodents.

#### 2.6.2. Reciprocal Crosstalk Between Oxidative Stress and Other Pathogenic Mechanisms

Oxidative stress synergizes with other pathogenic cascades involved in depression, as illustrated in Figure 4. Excessive ROS accumulation exacerbates glutamate excitotoxicity through enhancing spontaneous vesicular glutamate release or triggering reversed glutamate uptake [141, 142] and compromising glutamate reuptake via suppression of astrocytic GLT‐1 activity [139, 140] (Figure 4A1−3). Furthermore, LPS‐induced microglial glutamate release and IL‐1β/TNF‐α suppressed astrocytic glutamate reuptake were effectively abrogated by antioxidant butylated hydroxytoluene, NOX inhibitors, and nitric oxide synthase inhibitors, respectively [235, 257, 258]. These findings suggest that oxidative stress may serve as a prominent contributor to neuroinflammation‐driven glutamate excitotoxicity. In turn, excessive extracellular glutamate reciprocally exacerbates oxidative stress, through overactivation of extrasynaptic NMDA receptors and subsequent mitochondrial dysfunction [83] and disruption of the functional cooperation between GLT‐1 and xCT [138] (Figures 2A2/C1−3 and 4B1/2). Moreover, excessive ROS inhibit central *BDNF* expression, through directly suppressing CREB activity and enhancing the DNA binding affinity of NF‐κB [259, 260] (Figure 4A4) or indirectly potentiating glutamate excitotoxicity [84, 261] (Figure 2C4/6/7).

As the primary cellular source of ROS, mitochondria are, in turn, most vulnerable to excessive oxidative stress. Cumulative evidence demonstrates that mitochondrial dysfunction and restoration are closely related to the onset and remission of depression, respectively [262, 263]. For instance, CSDS‐triggered mitochondrial fission drove neuronal metabolic burden to promote stress susceptibility, while MS exposure or corticosterone administration elicited depressive‐like phenotypes by disrupting mitochondrial metabolic homeostasis [264–267]. Meanwhile, ATP, predominantly produced by mitochondria, has emerged as a crucial regulator of depressive pathogenesis [268]. Reduced ATP release from astrocytes in the PFC was tightly associated with increased susceptibility to CSDS‐induced depressive‐like behavior in mice, alleviated by exogenous ATP or stimulated endogenous ATP secretion [269, 270]. Further in‐depth mechanistic investigations are warranted to dissect the multifaceted roles of mitochondrial dysfunction in the depression pathophysiology. Furthermore, the reciprocal interplay between oxidative stress and the MGB axis constitutes a prominent regulatory mechanism in depression progression [271, 272]. In short, gut microbiota modulate ROS generation and antioxidant capacity through increasing the levels of indole‐containing molecules and short‐chain fatty acids (SCFAs) and enhancing intestinal NOX activity [273–275]. In turn, host mitochondrial genotypes and mutations shape gut microbiota diversity through ROS‐dependent signaling cascades [276]. In addition, excessive oxidative stress potentiates pro‐inflammatory cytokine expression via promoting AP‐1 and NF‐κB activity and enhancing histone acetylation, thereby exacerbating neuroinflammation [40, 277] (Figures 3D and 4A6).

### 2.7. MGB Axis Dysfunction

#### 2.7.1. MGB Axis Dysfunction in Depression

**Figure 5:**
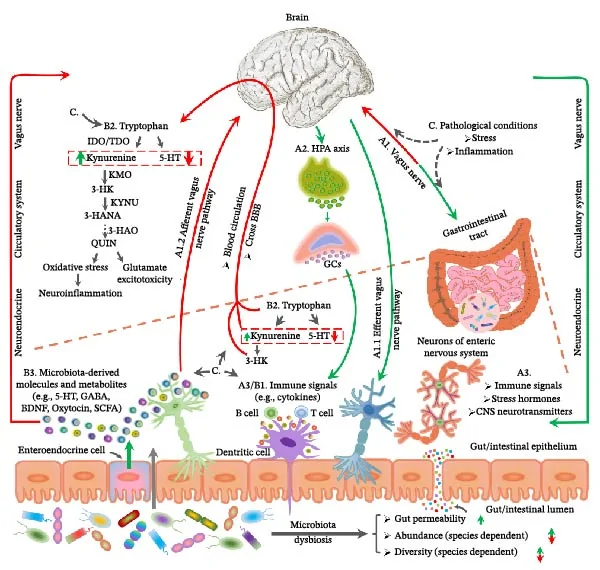
Involvement of MGB axis dysfunction in the pathogenesis of depression. The brain and gut (gastrointestinal tract) maintain bidirectional crosstalk, including top‐down brain‐to‐gut regulation (A) and bottom‐up gut‐to‐brain modulation (B). The brain modulates gut biological functions, including intestinal permeability and microbiota composition (abundance and diversity), predominantly via the efferent vagus nerve pathway (A1.1), neuroendocrine pathway (e.g., HPA) (A2), and circulatory immune signals, stress hormones, and CNS neurotransmitters (A3). In turn, bottom‐up gut‐to‐brain modulation is primarily mediated by immune signals (B1) and tryptophan metabolic reprograming (B2): pathological stimuli (e.g., stress and inflammation, C) suppress 5‐HT biosynthesis and promote kynurenine and QUIN generation by enhancing the enzymatic activities of IDO/TDO/KMO/KYNU/3‐HAO in both gut and brain tissues. Microbiota‐derived molecules and metabolites (e.g., 5‐HT and SCFAs) enter the brain via systemic circulation (B3) or target the afferent vagus nerve pathway (A1.2) to modulate central neuronal activity. 3‐HANA, 3‐hydroxyanthranilic acid; 3‐HAO, 3‐hydroxyanthranilic acid oxygenase; 3‐HK, 3‐hydroxykynurenine; 5‐HT, serotonin; BBB, blood‐brain barrier; BDNF, brain derived neurotrophic factor; CNS, central nervous system; GABA, gamma‐aminobutyric acid; GCs, glucocorticoids; HPA, hypothalamic–pituitary–adrenal; IDO, indoleamine 2,3‐dioxygenase; KMO, kynurenine 3‐monooxygenase; KYNU, kynureninase; MGB, microbial‐gut‐brain; SCFA, short chain fatty acid; TDO, tryptophan‐2,3‐dioxygenase.

The MGB axis represents reciprocal crosstalk between the brain and gastrointestinal tract via multiple pathways, such as neuroendocrine regulation (e.g., the HPA axis), tryptophan metabolism, and microbiota‐derived metabolites (e.g., SCFAs) [278–280] (Figure 5A1−3, B1−3). The vagus nerve and immune signals serve as the major bidirectional modulatory pathways. Top‐down brain‐to‐gut regulation (Figure 5A) modulates gut functions, including intestinal permeability and microbiota composition (abundance and diversity), through multiple pathways, such as the efferent vagus nerve pathway, neuroendocrine pathway (e.g., the HPA axis), and the circulatory system (Figure 5A1.1, A2/3). In turn, bottom‐up gut‐to‐brain modulation (Figure 5B) is primarily mediated by the afferent vagus nerve pathway, immune signals, reprogramed tryptophan metabolism, and microbiota‐derived molecules and metabolites (e.g., 5‐HT and SCFAs) (Figure 5B1−3, A1.2). Accumulating clinical and preclinical evidence underscores the pivotal implication of MGB axis dysfunction in depression pathogenesis, highlighting MGB axis modulation as a viable therapeutic strategy [22, 281]. Furthermore, cutting‐edge studies have further unraveled unprecedented mechanistic pathways underlying the brain‐gut interplay, which potentially participate in depression pathophysiology. Those emerging mechanisms encompass the microbiota–gut–immune–glia axis and 5‐HT^DRN^–ACh^DMV^–stomach axis [282, 283] and the peripheral trafficking of gut‐derived, microbiota‐specific T cells from the gut to the brain [284, 285]. Despite these research breakthroughs, the clinical translation of MGB axis‐targeted therapies still necessitates comprehensive and systematic preclinical and clinical investigations. Nevertheless, therapeutic strategies targeting the restoration of disrupted MGB axis homeostasis merit further exploration, such as probiotic supplementation (e.g., *Lactobacillus rhamnosus*), tryptophan enrichment, nutritional modulation, and administration of natural products with antidepressant properties (e.g., flavonoids, alkaloids, and polysaccharides) [286–289].

Chronic stress profoundly reshapes the microbiota composition (abundance and diversity) and the profile of microbiota‐derived molecules and metabolites. Furthermore, stress‐induced microbiota dysbiosis can be rescued via multiple strategies, ranging from conventional antidepressant regimens to microbiota‐modulating interventions such as probiotic supplementation and tryptophan enrichment. Clinically, depressed patients exhibited reduced abundance of *Bifidobacteria* and *Lactobacilli*, alongside increased abundance of *Bacteroidea*, *Proteus*, and *Actinobact eria* [281, 290]. Such microbial perturbations further interfere with the production of microbiota‐derived molecules and metabolites (e.g., 5‐HT, GABA, BDNF, oxytocin, and SCFAs), which modulate cerebral function by targeting the afferent vagus nerve pathway or crossing the BBB via systemic circulation [22] (Figure 5B3). A recent clinical study showed that stress‐induced microbiota dysbiosis led to amino acid deficiency, thereby increasing depression susceptibility in children and adolescents [291]. Consistently, rodent studies found that CSDS reduced *Tenericutes* and *Mollicutes*, whereas increased *Actinobacteria* and *Deltaproteobacteria* [292], or reduced *Bacteroidales*, *Clostridiales*, and *Mogibacteriaceae*, whereas increased *Ruminococaceae* [293]. Notably, stress‐triggered microbial remodeling might be dictated by the intrinsic functional properties of the gut microbiota: pro‐inflammatory detrimental taxa are selectively enriched, whereas anti‐inflammatory beneficial commensals are suppressed. In line with this notion, depressed individuals exhibited overabundant pro‐inflammatory bacteria (e.g., *Enterobacteriaceae*) and depleted SCFAs producing bacteria (e.g., *Prevotellaceae*) [294]. Furthermore, pharmacological interventions with antidepressants effectively reverse these microbial abnormalities: both (R)‐ketamine and (S)‐ketamine significantly normalized CSDS‐increased abundance of *Deltaproteobacteria* and reduced abundance of *Bacteroidales* and *Clostridiales*, in susceptible mice, respectively [292, 293]. Additionally, shifts in microbial composition and metabolic profiles modulated the antidepressant response of CUMS‐treated mice to escitalopram, indicating that microbial signatures mediate antidepressant therapeutic efficacy [295].

The modulatory effects of chronic stress and antidepressant interventions on gut microbiota composition are highly taxonomic rank‐dependent across phylum, class, order, family, genus, and species. For instance, CSDS reduced *Tenericutes* while increasing *Actinobacteria* at the phylum level [292]. Furthermore, CSDS reduced *Bacteroidales* and *Clostridiales* at the order level, whereas suppressed *Mogibacteriaceae* yet upregulated *Ruminococaceae* at the family level [293]. Regarding antidepressant‐mediated regulation, (R)‐ketamine effectively normalized CSDS‐triggered microbial alterations at both order and family levels [293]. In contrast, neither (R)‐ketamine nor (S)‐ketamine exerted prominent regulatory effects on CSDS‐elicited bacterial alterations at the phylum level [292]. It is worth noting that only (R)‐ketamine rescued CSDS‐induced decrease in *Mollicutes*, whereas both ketamine enantiomers attenuated CSDS‐triggered upregulation of *Deltaproteobacteria* at the class level [292]. Consistently, microbiota‐targeted interventions exert prominent antidepressant efficacy, highlighting the pivotal contribution of MGB axis dysfunction from another perspective. For instance, probiotic supplementation with *Bifidobacterium* and *Lactococcus lactis* effectively rescued CUMS‐ or MS‐induced depressive‐like behavior and, moreover, conferred host resilience to CSDS‐elicited depressive‐like phenotypes in rats and mice, respectively [296–298]. In parallel, the urease‐producing strain *Streptococcus thermophilus* ameliorated CSDS‐elicited depressive‐like behavior in mice, indicating a critical modulatory role of the microbiota in host susceptibility to chronic stress [299].

Notably, FMT studies provide convincing evidence demonstrating that disrupted MGB axis homeostasis contributes to depression etiology, while restoring its homeostasis serves as a viable therapeutic strategy. On the one hand, FMT using samples from depressed patients [300–302] or CUMS‐exposed animals [303, 304] was sufficient to induce robust depressive‐like behavior in recipient mice and rats, respectively. On the other hand, FMT from healthy donors alleviated depressive phenotypes in both clinical patients and preclinical depressive models [305, 306]. Despite these promising observations, clinical translation of FMT for depression treatment remains in its infancy. Further rigorous investigations are still warranted to screen antidepressant‐prone microbial trains, optimize targeted and efficient delivery strategies, and systematically validate the long‐term safety and therapeutic efficacy in clinical practice [307].

#### 2.7.2. Reciprocal Crosstalk Between MGB Axis Dysfunction and Other Pathogenic Mechanisms

Dysregulated MGB axis homeostasis interferes with central neurotransmission, particularly cerebral 5‐HT signaling. Although gut‐synthesized 5‐HT cannot penetrate the BBB, circulating tryptophan and its downstream metabolites, including kynurenine and 3‐hydroxykynurenine (3‐HK), possess the capacity to cross the BBB (Figure 5B2). Such molecular permeability constitutes a vital peripheral‐to‐central pathway, whereby the gut microbiota remotely modulates diverse cerebral functions. Under pathological conditions (e.g., stress and inflammation), KP is excessively promoted, thereby substantially compromising central 5‐HT biosynthesis [49, 278] (Figures 3A4/5 and 5B2/C). From a therapeutic perspective, tryptophan metabolism‐targeted strategies, such as supplementation with probiotic *Bifidobacterium*, exogenous tryptophan, and melatonin, effectively ameliorated depressive‐like behavior in rats and mice, respectively [55, 308–310]. Such therapeutic effects are primarily ascribed to the restructured gut microbiota and restored central 5‐HT signaling [51, 229]. Beyond 5‐HT signaling, CUMS also induced pervasive perturbations in central BDNF, glutamatergic, and GABAergic signaling, all of which were effectively reversed by tryptophan supplementation and probiotic interventions (e.g., *Lactococcus lactis* and *Bifidobacterium*) [55, 298, 311, 312]. Additionally, glutamate derived from gut‐residing bacterium *Anaerotruncus colihominis* exacerbated depressive‐like phenotypes in mice [313], whereas ketamine rectified microbiota dysbiosis and consequently normalized aberrant glutamatergic and GABAergic signaling [314].

Apart from aberrant neurotransmitter signaling, MGB axis dysfunction interacts closely with neuroinflammation, oxidative stress, and mitochondrial dysfunction, which collectively exacerbate depression progression [315–317]. Cumulative evidence indicates that microbiota dysbiosis exerts pro‐depressant effects by eliciting neuroinflammation, whereas microbiota homeostasis restoration relieves neuroinflammation and confers antidepressant benefits. On the one hand, CUMS exposure and microbiota dysbiosis induced by FMT from CUMS‐exposed animals markedly increased the expression of NLRP3 and IL‐1β in the hippocampus of recipient mice and rats, accompanied by remarkable depressive‐like phenotypes [202, 304]. On the other hand, restoring MGB axis homeostasis via multiple therapeutic approaches, including probiotic supplementation, FMT using healthy donor‐derived microbiota, and targeted anti‐inflammatory therapy, produces robust antidepressant efficacy by suppressing neuroinflammation. Specifically, supplementation with the probiotic *Clostridium butyricum* or FMT using the microbiota from healthy donors effectively mitigated depressive‐like behavior in mice [302, 318]. Likewise, probiotic intervention effectively reversed CUMS‐induced pro‐inflammatory and pro‐depressive alterations [304]. Consistently, rifaximin treatment ameliorated CUMS‐triggered depressive‐like phenotypes in rats by recovering SCFA synthesis and restoring depleted populations of *Ruminococcaceae* and *Lachnospiraceae* [52, 319].

## 3. Discussion and Future Perspectives

Depression constitutes an escalating threat to global mental health and represents a prevalent, intractable public health challenge that necessitates timely and effective therapeutic interventions. Over the past decades, accumulating clinical observations and preclinical investigations, predominantly based on chronic stress rodent models, have substantially deepened our mechanistic understanding of depression, fostered the establishment of multiple hypotheses, and accelerated the development of first‐line antidepressant strategies. This review systematically recapitulates prevailing mechanistic hypotheses, primarily encompassing the classic theories regarding HPA axis dysregulation and 5‐HT deficiency, intermediate mechanisms centered on glutamate excitotoxicity and BDNF/TrkB dysregulation, and emerging theories of neuroinflammation, oxidative stress, and MGB axis dysfunction. We aim to dissect their multifaceted neurobiological underpinnings, with a particular emphasis on sophisticated molecular mechanisms mainly uncovered by preclinical research using chronic stress‐induced rodent models. Furthermore, we elucidate the intricate crosstalk and bidirectional regulation among these mechanistic pathways. It is worth noting that other pathogenic mechanisms of depression have been identified, such as genetic predisposition [320], epigenetic modifications [321], impaired hippocampal neurogenesis [322], and disrupted neuron–astrocyte coupling [323]. These topics are not addressed in the present review, given the incomplete relevant theoretical framework and limited manuscript scope.

### 3.1. Limitations of Current Pathogenesis Hypotheses and Antidepressants

Despite substantial advances to date, each prevailing pathogenic hypothesis and corresponding antidepressant agent possess inherent limitations and undesirable side effects, which constitute major obstacles hindering the bench‐to‐bedside translation of preclinical discoveries. Although HPA axis dysregulation has been well recognized for decades, therapeutic strategies designed to restore HPA axis homeostasis, such as modulating GR expression or sensitivity with agonists or sensitizing agents, fail to produce satisfactory clinical outcomes. This translational gap is largely attributable to heterogeneous HPA axis alterations (hyper‐ or hypocortisolism) and individual heterogeneity (e.g., genetic predisposition, sex disparities, and age‐dependent discrepancies). The monoamine deficit theory has laid the fundamental groundwork for the development of first‐generation and subsequent conventional monoamine‐targeted antidepressants, such as TCAs, MAOIs, and SSRIs. Nevertheless, these conventional pharmacological agents are plagued by two major limitations: delayed therapeutic onset and inadequate efficacy against TRD. In contrast, glutamate‐modulating antidepressants (e.g., esketamine) exert rapid antidepressant actions and robust efficacy against TRD, with a single dose sustaining therapeutic benefits for approximately 2 weeks. Nevertheless, the US Food and Drug Administration only approves esketamine for treating patients diagnosed with TRD and major depressive disorder accompanied by severe suicidal ideation. This restricted approval stems from its addictive potential and diverse adverse reactions, most notably psychiatric disturbances and dissociative symptoms. Furthermore, given the ubiquitous distribution of glutamatergic signaling across the entire brain, precisely restoring region‐specific glutamate homeostasis while eliminating off‐target toxicities remains a major challenge and obstacle for clinical translation. Regarding BDNF‐targeted antidepressant regimens, their clinical applicability is severely constrained by two intrinsic biological traits: BDNF possesses an extremely short half‐life, ~1 min in plasma and 1 h in the CSF, whereas depression‐related BDNF dysregulation exhibits distinct brain region‐specific patterns. Notably, conventional systemic delivery routes for antidepressants, including oral administration and nasal delivery, compromise the targeting accuracy and therapeutic efficacy of BDNF‐targeted interventions. In terms of anti‐inflammatory and antioxidant therapeutic strategies, such approaches typically exert robust antidepressant effects only in specific depressive subtypes driven by neuroinflammation and oxidative stress. Furthermore, neuroinflammation and oxidative stress constitute systemic pathological perturbations, which trigger extensive biological dysfunction via activating multiple downstream signaling cascades and driving a broad range of pathological impairments. One pivotal depression‐relevant outcome is disrupted neurotransmission, manifested as dysregulated 5‐HT, glutamate, and BDNF signaling as described above. Notably, such aberrant signaling often fails to restore physiological homeostasis, even following targeted anti‐inflammatory or antioxidant interventions. Regarding MGB axis dysfunction, two major challenges currently impede in‐depth mechanistic elucidation and subsequent clinical translation. First, depression‐associated alterations of the microbiota composition and microbiota‐derived metabolites are highly heterogeneous, rendering accurate identification and quantification rather difficult. Furthermore, given the limitations of current research methodologies, it remains considerably challenging, if not currently unfeasible, to establish a definitive one‐to‐one causal link between specific microbiota species and discrete central neurotransmission. Additionally, both the gut microbiota and cerebral neurotransmitters undergo highly dynamic developmental trajectories, which are constantly shaped by multiple internal and external variables, such as dietary patterns and emotional fluctuations. Despite these persistent challenges and inherent uncertainties, therapeutic strategies targeting MGB axis homeostasis restoration still remain a promising direction for future antidepressant development.

### 3.2. Emerging Targets and Future Directions for Research

Ongoing investigations into depression pathogenesis and therapeutic strategies constantly yield novel and insightful findings. Recent preclinical rodent studies have unraveled the essential mediating role of neuron‐astrocyte coupling in driving stress‐related pathological changes [323], rapid modulation of gut microbiota by hypothalamic circuits [324], and sustained antidepressant effect of ketamine mediated by enhanced ERK activity [325]. Compelling evidence further shows that multiple potassium channels (e.g., KCNQ Kir4.1 and KV7) serve as pivotal regulators of depression pathogenesis, thereby supporting channel‐targeted modulation as a feasible and promising therapeutic option [326–328]. In line with preclinical evidence, a recent clinical trial showed that azetukalner, a novel KV7 potassium channel opener, elicited potent antidepressant efficacy [329]. Additionally, emerging studies have uncovered previously unrecognized functions of classic monoaminergic signaling and clarified how non‐monoaminergic antidepressants modulate monoaminergic transmission. Representative evidence includes norepinephrine‐induced ATP release from astroglial cells [330], norepinephrine‐driven astrocyte‐dependent synaptic modulation [331], potent therapeutic efficacy of dopaminergic agonist pramipexole against TRD [332], ketamine‐elicited neural plasticity within norepinephrine‐astroglial circuits [330], and ketamine‐mediated regulation of D1 dopamine receptor activity in the NAc [46]. Collectively, these groundbreaking findings substantially broaden our current mechanistic comprehension, accelerate the development of innovative antidepressant strategies, and optimize conventional therapeutic regimens.

## 4. Conclusions

In conclusion, to construct a holistic landscape of depression pathogenesis, this review systematically recapitulates prevailing pathogenic hypotheses and associated multifaceted neurobiological underpinnings, with key evidence predominantly obtained from preclinical chronic stress rodent models. We outline the defining features of each mainstream hypothesis and the intricate crosstalk and bidirectional regulatory networks among distinct mechanistic pathways, focusing on underlying molecular signaling cascades. Thoroughly unraveling the multilayered pathogenic mechanisms of depression and translating them into optimized, patient‐tailored therapeutic regimens remain challenging endeavors. Although substantial obstacles persist and sustained iterative investigation is indispensable, each incremental advance steadily narrows the gap toward conquering depression. As an ancient Chinese proverb states: The road ahead may be rugged and lengthy, yet the destination awaits those who forge ahead; tasks may be arduous, yet persistence will yield eventual success.

## Author Contributions

Bo Zhang and Chuanyu Li designed the figures, wrote, edited, and revised the manuscript.

## Funding

This work was supported by the National Natural Science Foundation of China (Grant 32260200), Guangxi Natural Science Foundation (Grants 2026GXNSFHA00640017, 2025GXNSFAA069126, and 2020GXNSFAA297134), and Guangxi Science and Technology Base and Talent Special Project (Grant GuiKe‐AD20238023).

## Disclosure

All authors have read and approved the publication of the manuscript. The authors take full responsibility for the entire content of the manuscript.

## Conflicts of Interest

The authors declare no conflicts of interest.

## Data Availability

No data were presented in the manuscript.
